# National Mental Health Platform in Egypt, Revolution of Mental Health Services

**DOI:** 10.1192/j.eurpsy.2022.1471

**Published:** 2022-09-01

**Authors:** S. Noby

**Affiliations:** General Secretariat of Mental Health and Addiction treatment, Ministry of Health, Research Unit, Cairo, Egypt

**Keywords:** Egypt, health, digital, Mental

## Abstract

**Introduction:**

The General Secretariat of Mental Health and Addiction Treatment is a governmental body dedicated to the provision of mental health services in Ministry of Health in Egypt. Moreover, in response to the COVID-19 outbreak in Egypt, on line, advice tips, and counseling through the hotline services have became available through social media sites the past few months. Such digital remote MH services were very much welcomed by social media users. In the same time, for mental health professionals, the opportunity to provide help in this time of crisis – without an in-person consultation – was very desirable. In addition to the prevailing stigma of MH condition in the Egyptian culture, which this remote approach overcome it. Effective innovations in the field of mental health have the potential to change the face of mental health care, not only during pandemics but also in routine daily life.

**Objectives:**

1. Develop a National strategy for E-Mental Health in Egypt. 2. Develop the National mental health platform as a universal implementation tool.

**Methods:**

This project conducted in collaboration with WHO in Egypt, It run in 3 phases: Strategy, planning and infrastructure: include needs assessment and software development. Finalizing infrastructure, adaptation of the content Deployment: include training of the service providers on the usage of the adapted M.H platform, launching and advocacy.

**Results:**

Development of E-Mental Health strategy, Development of National Mental Health Platform, Training of 50 therapists on digital mental health services.

**Conclusions:**

National Mental Health Platform is the future road of mental health services in Egypt

**Disclosure:**

No significant relationships.

